# Adherence to the MIND diet and the odds of mild cognitive impairment in generally healthy older adults: The 3-year DO-HEALTH study

**DOI:** 10.1016/j.jnha.2023.100034

**Published:** 2024-02-05

**Authors:** Roman Sager, Stephanie Gaengler, Walter C. Willett, E. John Orav, Michele Mattle, Jana Habermann, Katharina Geiling, Ralph C. Schimmer, Bruno Vellas, Reto W. Kressig, Andreas Egli, Bess Dawson-Hughes, Heike A. Bischoff-Ferrari

**Affiliations:** a Centre on Aging and Mobility, University of Zurich, Zurich, Switzerland; b Department of Geriatrics and Aging Research, University of Zurich, Zurich, Switzerland; c Department of Nutrition, Harvard T.H. Chan School of Public Health, Boston, MA, USA; d Department of Epidemiology, Harvard T.H. Chan School of Public Health, Boston, MA, USA; e Department of Biostatistics, Harvard T.H. Chan School of Public Health, Boston, MA, USA; f University Clinic for Aging Medicine, Zurich City Hospital — Waid, Zurich, Switzerland; g Department of Aging Medicine and Aging Research, University Hospital Zurich and University of Zurich, Zurich, Switzerland; h Gérontopôle de Toulouse, Institut du Vieillissement, Centre Hospitalo-Universitaire de Toulouse, Toulouse, France and UMR INSERM 1027, University of Toulouse III, Toulouse, France; i IHU HealthAge, University Hospital Toulouse, France; j University Department of Geriatric Medicine FELIX PLATTER and University of Basel, Basel, Switzerland; k Jean Mayer USDA Human Nutrition Research Center on Aging, Tufts University, Boston, MA, USA

**Keywords:** Cognitive decline, Prevention, Inflammaging, Dietary patterns, Reliable change index, Practice effect

## Abstract

**Background:**

The Mediterranean-DASH Intervention for Neurodegenerative Delay (MIND) diet may slow cognitive decline in older adults. A potential mechanism could be possible anti-inflammatory properties of the MIND-diet.

**Objective:**

To examine whether adherence to the MIND diet at baseline is associated with the odds of mild cognitive impairment (MCI) and changes in biomarkers of inflammation (High-sensitivity C-reactive Protein(hsCRP), interleukin-6(IL-6)) over three years in adults ≥70 years.

**Methods:**

Adherence to the MIND diet was assessed by food frequency questionnaire (FFQ) at baseline and after three years. Presence of MCI based on the Montreal Cognitive Assessment (MoCA) was defined as <26 (MCI_26_), or <24 (MCI_24_). We performed a minimally adjusted model controlling for sex, prior fall, linear spline at age 85, time, treatment and study site. The fully adjusted model also adjusted for education, BMI, physical activity, depression score, daily energy intake, and comorbidity score. To assess the change in inflammatory markers from baseline, we used linear-mixed-effect models adjusted for the same variables plus the respective baseline concentrations. Sensitivity analyses accounting for practice effects of repeated cognitive tests using the reliable change index for both MoCA cut-offs were done.

**Results:**

We included 2028 of 2157 DO-HEALTH participants (60.5% women; mean age 74.88 years) with complete data. Adherence to the MIND diet at baseline was not associated with cognitive decline over three years, neither at MoCA < 26 (OR (95%CI) = 0.99 (0.94–1.04)) nor at MoCA < 24 (OR (95%CI) = 1.03 (0.96–1.1)). Applying the reliable change index to the two cut-offs confirmed the findings. Further, the MIND diet adherence was not associated with the change in MoCA score from baseline in DO-HEALTH. For inflammatory biomarkers MIND-diet baseline adherence was not associated with changes in hsCRP or IL-6.

**Conclusion:**

Adherence to the MIND-diet was neither associated with the odds of MCI, nor with hsCRP or IL-6 at baseline. Moreover, change in MIND-diet over three years was not associated with changes in hsCRP or IL-6.

## Highlights

• MIND diet was not associated with odds of MCI.
• MIND diet was not associated with hsCRP or IL-6.
• Practice effect should be accounted for in repeated assessment of cognitive tests.

## 1 Introduction

The World Health Organization expects the number of patients with dementia to triple from 50 million people in 2015 to 152 million by 2050 [1]. Recent research suggests that up to 40% of dementia cases may be prevented with healthy lifestyle choices, which is of pivotal importance for public health [2]. Healthy nutrition [3,4], such as plant-based diets, may protect from cognitive decline based on their high content in antioxidants [5], polyphenols [6], and polyunsaturated fats [7] of plant-based foods. Conversely, animal-based foods, high in saturated fat or trans-fats, may accelerate cognitive aging [4,8].

The MIND-diet has been specifically designed to delay neurodegenerative processes [9]. It focuses on the consumption of ten food groups that are considered brain-healthy and the reduction of five food groups, which are considered unhealthy for cognitive performance [[10], [11], [12]]. Morris et al. found that among participants within the highest tertile of the MIND score, the incidence rate of Alzheimer’s disease was 53% reduced compared to participants in the lowest adherence tertile [9,13]. To date, some [9,14,15] but not all [16,17] studies found that a higher adherence to the MIND diet is associated with a reduction in cognitive decline.

Regarding mechanistic pathways of the MIND diet, it has been suggested that the increased intake of antioxidants [5], polyphenols [6], polyunsaturated fats [7] and simultaneously reduction of foods high in saturated fats or trans-fats [4,8], may reduce inflammation and thereby be neuroprotective. In fact, inflammatory processes have been suggested to be early triggers in AD pathogenesis [18,19] and an increased inflammatory state has been consistently associated with higher risk for Alzheimer’s disease [20]. Two widely used biomarkers for inflammation are the high sensitivity C-reactive protein (hsCRP) and interleukin-6 (IL-6) [21,22]. To the best of our knowledge, however, no prior study assessed the association between adherence to the MIND diet with blood levels of hsCRP and IL-6.

In this analysis, we take advantage of the DO-HEALTH clinical trial, which, among other outcomes, assessed cognitive function and food intake in 2157 generally healthy and active adults aged 70 and older at baseline and over a 3-year follow-up. Our aims are to investigate the association between adherence to the MIND diet and the odds of MCI based on the MoCA questionnaire and using two cut-offs for its score to define MCI as suggested in the literature [23,24]. Further, we aim to investigate if a MIND diet adherence is associated with a change in low-grade chronic inflammation measured by blood levels of hsCRP and IL6.

## 2 Material and methods

### 2.1 Study design and participants

This is an observational prospective analysis of the 3-year DO-HEALTH clinical trial. DO-HEALTH is a multi-center, double blind, randomized controlled clinical trial designed to support healthy aging in European older adults [25]. The trial examined the individual and combined effects of Omega-3, Vitamin-D and Simple Home Exercise over 3 years of follow-up. A total of 2157 community-dwelling men and women aged 70 years and older were recruited from seven centers in five European countries, in particular Zurich, Basel, Geneva, Berlin, Innsbruck, Toulouse, and Coimbra. Further inclusion criteria were a Mini-Mental State Examination score ≥24, no major health event in the past five years before inclusion, and sufficient mobility to visit the study centers. The median follow-up was 2.99 years [26]. All participants provided written consent to participate in DO-HEALTH and the further use of their data [26]. The ethics committee of the canton of Zurich approved the further use of data within this study (BASEC Nr. 2021-00913).

### 2.2 Cognitive assessment and reliable change index

The Montreal Cognitive Assessment (MoCA) was assessed in a standardized way across all DO-HEALTH centers, in person, and by trained and DO-HEALTH-certified healthcare professionals at baseline and at each yearly visit over 3 years during the DO-HEALTH trial. The MoCA was originally designed and validated to assess MCI rather than continuous cognitive decline, therefore we defined mild cognitive impairment (MCI) as MoCA score <26 or <24, as both cut-offs are supported in the literature [23,24].

Additionally, we calculated the reliable change index (RCI), applied to both MoCA cutoffs as a sensitivity analysis. The RCI is a statistical method proposed by Chelune et al. and Perdices et al. to control for practice effects in cognitive tests [27,28]. The RCI was calculated in the placebo group of the DO-HEALTH trial to avoid any potential treatment effects. To do so, the formula suggested by Chelune et al. [27] was applied to calculate the 90% confidence interval of the standard error of the measurement difference σ_DIFF_. The largest confidence interval generated at the three follow-up time points was used as most conservative thresholds to determine the MCI status at each time point, namely −2 and +4 points. The baseline assessment was the reference. If participants improved 4 points at one time point and managed to go from impaired to normal then this was counted as true improvement, else the participant remained at the same diagnosis as the previous assessment. Vice versa if a participant decline by 2 points from one assessment to the next and fell below the respective threshold for MCI the decline was counted a true decline, else the participant remained with the status as the previous assessment.

$$\sigma_{DIFF}=\left[\sigma_{x}\sqrt{2\left(1-r_{XY}\right)}\right]-PE$$

σ_X_ = standard deviation of the test score at time 1

r_xy_ = test-retest correlation

PE = practice effect (difference of the mean at baseline and the mean in the follow-up measurement in the placebo group)

### 2.3 Dietary assessment

At baseline and year three, participants completed the 216-item DO-HEALTH food frequency questionnaire (FFQ) created in collaboration with the Department of Nutrition at Harvard School of Public Health for European older adults [25].

The MIND Score was calculated based on ten food groups which are considered brain healthy (green leafy vegetables, other vegetables, whole grains, beans, nuts, berries, wine, poultry, olive oil and fish) and five food groups considered unhealthy for cognitive function (red meat, butter and margarine, cheese, pastries and sweets and fried foods) [9]. The adherence was calculated based on predefined ideal intake frequencies for each of the fifteen food groups. The total score lies between 0 and 15 with higher score indicating better adherence to the MIND diet. This calculation is in accordance with the original calculation proposed by Morris et al. [9].

### 2.4 Inflammatory biomarkers

Fasting blood samples were collected at baseline and at each yearly visit of the DO-HEALTH study. They were processed immediately and stored at −80 °C until analysis. All blood biomarkers were analyzed at Institute for Clinical Chemistry at the University Hospital Zurich, Switzerland (KCI, ISO Certified ISO/IEC 17025) under standardized procedures and with regular quality checks. Concentrations of hsCRP and IL-6 were measured in plasma, using heparin tubes. HsCRP was measured using a particle enhanced immunological turbidity assay on a Roche Cobas 8000 analyzer with a c701 module with a limit of detection (LOD) of 0.3 mg/L. The calibrated range was 0.3–350 mg/L. The coefficients of variation for the low- and high-concentration quality control samples were 2.4% and 2.8%, respectively.

IL-6 was measured using an electro chemiluminescence assay on a Roche Cobas c602 with a LOD of 1.5 ng/L. Values below the LOD were imputed with half the LOD. The calibrated range was 1.5–5000 ng/L. The coefficients of variation for the low- and high-concentration quality control samples were 2.7% and 1.9%, respectively.

### 2.5 Statistical analysis

The data were assessed for their distribution using histograms and quantile plots. Normally distributed data are presented as mean and standard deviation, and non-normally distributed data as median and interquartile range. Categorical data are presented as frequency and percentage. Differences between tertiles of MIND diet score were tested using ANOVA, Kruskal–Wallis test and chi-square test for normal, non-normal and categorical variables, respectively.

The longitudinal association between odds of MCI and baseline MIND diet score over three years was assessed using generalized estimating equations for repeated binary outcomes and unstructured covariance. We performed a minimally adjusted model for sex, treatment, treatment *time, time, prior fall, linear spline at age 85, study site, and baseline MoCA score. The fully adjusted model was additionally adjusted for years of education, Body Mass Index (BMI), total self-reported physical activity, geriatric depression scale 15 (GDS-15), energy intake, and Sangha–comorbidity score. The adjustments have been determined based on the trial design and previous published studies, the composition of a minimally adjusted and fully adjusted model have been determined with DAGitty [29]. In addition we assessed the effect of the MIND diet on the change in continuous MoCA score from baseline in a linear mixed effect model accounting for repeated measures, adjusted for the same covariates as named for the change in the binary score.

The association between change in inflammatory biomarkers from baseline with baseline MIND diet score was assessed using linear mixed effect models with a random intercept for participants. Both minimally and fully adjusted models were run using the same adjustments as mentioned above.

Subgroup analyses by sex and education years (≤12 or >12 years) were performed if the interaction of the subgroup variable and the exposure had a p < 0.05.

All analyses were performed in R (R 4.1.1; RStudio 2022.02.2+485).

## 3 Results

This prospective observational analysis of the DO-HEALTH study was conducted using 2028 out of the original 2157 community dwelling adults aged ≥70 years. We excluded participants with daily energy intakes exceeding 4000 kcal and 5000 kcal (n = 123) for women and men, respectively. We further excluded participants with >10% missing values in the FFQ (n = 6). The mean age among the 2028 participants was 74.88 (±4.42) years, 60.5% were women. Mean BMI was 26.27 (±4.25) kg/m^2^ and the average years of education was 12.72 (±4.27) years. Participants in the highest tertile of the MIND diet score were more likely to be women (69.2%), tended to have comparatively fewer years of education, had a lower alcohol intake, and were more likely to live alone (Table 1). The frequency of MCI cases based on the different cut-offs applied in this paper can be found in **Table S1** in the Supplement.

**Table 1:** Baseline characteristics of the analyzed DO-HEALTH population by tertiles of the MIND diet score.

Baseline characteristics	MIND diet				p-Value
	Tertile 1	Tertile 2	Tertile 3	Overall	
MIND diet score, range	3–7.5	8–9	9.5–13	–	
Total (n)	753	727	548	2028	
Women, n (%)	410 (54.4)	437 (60.1)	379 (69.2)	1226 (60.5)	<0.001
Age, years, mean (SD)	75.07 (4.62)	74.80 (4.23)	74.73 (4.36)	74.88 (4.42)	0.316
Body Mass Index (BMI), kg/m^2^, mean (SD)	26.47 (4.24)	26.13 (4.22)	26.16 (4.30)	26.27 (4.25)	0.244
Total Energy Intake, kcal/day, mean (SD)	2423.38 (717.62)	2504.75 (721.22)	2586.45 (733.98)	2496.61 (725.90)	<0.001
Metabolic equivalent of task (MET), MET-h/week, median [IQR]^a^	26.17 [11.30, 52.88]	28.38 [12.19, 54.79]	25.75 [12.50, 47.54]	26.67 [11.91, 52.03]	0.281
Physical activity, n (%)					0.400
None	138 (18.3)	115 (15.9)	101 (18.4)	354 (17.5)	
1−2 times per week	239 (31.7)	222 (30.6)	155 (28.3)	616 (30.4)	
≥3 times per week	376 (49.9)	388 (53.5)	292 (53.3)	1056 (52.1)	
Education, years, mean (SD)	12.98 (4.04)	12.76 (4.05)	12.31 (4.83)	12.72 (4.27)	0.021
Level of education, n (%)					<0.001
Basic	36 (4.8)	45 (6.3)	72 (13.3)	153 (7.6)	
Lower	128 (17.2)	116 (16.1)	88 (16.3)	332 (16.6)	
University	114 (15.3)	140 (19.5)	105 (19.4)	359 (17.9)	
Upper	151 (20.3)	141 (19.6)	128 (23.7)	420 (21.0)	
Vocational	316 (42.4)	277 (38.5)	147 (27.2)	740 (36.9)	
Living alone, n (%)	294 (39.0)	286 (39.3)	254 (46.4)	834 (41.1)	0.014
Sangha score, median [IQR]^a^	3.00 [1.00, 5.00]	2.00 [1.00, 5.00]	3.00 [1.00, 5.00]	3.00 [1.00, 5.00]	0.784
GDS15-score, median [IQR]^a^	1.00 [0.00, 2.00]	1.00 [0.00, 2.00]	1.00 [0.00, 3.00]	1.00 [0.00, 2.00]	0.045
MoCA-score, median [IQR]^a^	26.00 [24.00, 28.00]	26.00 [24.00, 28.00]	26.00 [24.00, 28.00]	26.00 [24.00, 28.00]	0.122
Alcohol consumption, g/day, median [IQR]^a^	4.31 [0.83, 11.79]	5.22 [1.33, 12.28]	5.06 [0.83, 11.77]	5.06 [0.84, 11.79]	0.029
Interleukin-6, ng/L, median [IQR]^a^	2.60 [1.70, 4.00]	2.50 [1.60, 3.90]	2.50 [1.50, 3.70]	2.50 [1.60, 3.90]	0.483
High-sensitivity C-reactive protein, mg/L, median [IQR]^a^	1.50 [0.90, 3.20]	1.50 [0.80, 2.90]	1.50 [0.80, 2.80]	1.50 [0.80, 3.00]	0.242

*Abbreviations*: SD = standard deviation, IQR = interquartile range, MET = metabolic equivalent of task.

a A Kruskal–Walis test was performed because the collected data does not follow a normal distribution.

### 3.1 Baseline MIND diet adherence

For the MoCA MCI cut-off of <26 (MCI_26_), baseline MIND score was not associated with MCI_26_, either in the minimally or the fully adjusted model (Table 2). Also, for the MoCA MCI cut-off of <24 (MCI_24_), baseline MIND score was not associated with MCI_24_, in the minimally or the fully adjusted model (Table 2). These results remained unchanged after adjusting for practice effect for both the <26 and <24 MCI MoCA cutoff (Table 2).The MIND score was not associated with the change in MoCA score from baseline (Table 3).

**Table 2:** Longitudinal analysis over 3 years of the association of the MIND score with cognitive impairment evaluated at a MOCA score <26 and <24. Sensitivity analysis includes adjustment for reliable change index (RCI) to account for practice effect.

Exposure	Model	MoCA < 26		MoCA < 24		MoCA < 26 RCI		MoCA < 24 RCI	
		OR (95%CI)	p-Value	OR (95%CI)	p-Value	OR (95%CI)	p-Value	OR (95%CI)	p-Value
Baseline MIND score	Minimal	0.97 (0.92–1.03)	0.317	1.00 (0.94–1.07)	0.91	0.99 (0.92–1.05)	0.673	0.99 (0.91–1.07)	0.781
	Full	0.99 (0.94–1.04)	0.62	1.03 (0.96–1.1)	0.426	1.00 (0.94–1.07)	0.917	1.01 (0.93–1.1)	0.848

*Abbreviations*: MOCA = Montreal cognitive assessment, OR = odds ratio, CI = confidence interval, RCI = reliable change index.

Basic adjusted model adjusted for: sex, treatment, time, treatment *time, prior fall, age, linear spline at age 85, study site, baseline MoCA score.

Fully-adjusted model adjusted for: basic model plus education, body mass index (BMI), physical activity, geriatric depression scale (GDS) 15, energy intake, Sangha score, current smoker.

**Table 3:** Longitudinal analyses of the 3-year changes in MoCA score and inflammatory markers with the MIND diet score.

Exposure	Model	Change in MoCA from baseline		Change in IL-6 [ng/L]		Change in hsCRP [mg/L]	
		Beta-coefficient ± SE	p-Value	Beta-coefficient ± SE	p-Value	Beta-coefficient ± SE	p-Value
Baseline MIND score	Minimal	0.03 + −0.03	0.254	0.06 ± 0.04	0.132	−0.05 ± 0.04	0.201
	Full	0.01 + −0.03	0.790	0.06 ± 0.04	0.177	−0.04 ± 0.04	0.304

*Abbreviations*: MOCA = Montreal cognitive assessment, SE = standard error.

Basic adjusted model adjusted for: sex, treatment, time, treatment *time, prior fall, age, linear spline at age 85, study site, baseline MoCA score/inflammatory marker concentration.

Fully-adjusted model adjusted for: basic model plus education, body mass index (BMI), physical activity, geriatric depression scale (GDS) 15, energy intake, Sangha score, current smoker.

### 3.2 MIND diet and change in inflammatory biomarkers

Adherence to the MIND diet at baseline was not associated with the change in hsCRP or IL-6 from baseline over three year (Table 3).

There was no significant interaction between the MIND diet adherence and pre-defined subgroup variables, therefore no subgroup analyses were conducted.

## 4 Discussion

In this prospective study of over 2000 generally healthy and active adults aged 70 years and older, we aimed to assess the association of adherence to the MIND-diet and the odds of mild cognitive impairment (MCI) measured by the MoCA questionnaire. Baseline MIND-diet adherence was not associated with the odds of MCI with neither cut-off (MCI_26_ and MCI_24_). When accounting for the practice effect, the results remained unchanged. Moreover, there was no significant association between MIND diet adherence and the inflammatory markers IL-6 and hsCRP.

Our findings are to some extent consistent with previous findings. A recently published randomized controlled trial by Barnes et al. found no significant association between MIND diet intervention and changes in global cognition over three years compared to a control diet in people with a suboptimal diet and a family history of dementia [30].

On the other hand, a recently published systematic literature review by Kheirouri et al. found significant association between better adherence to the MIND-diet and better global cognitive performance in seven out of nine longitudinal studies analyzed [15]. Some of the heterogeneity regarding the benefit of adherence to the MIND diet in the studies described above may be explained by the findings of the Rotterdam cohort, where an initial association between the adherence to the MIND-diet and decreased risk of dementia disappeared after seven years of the 15.6 years follow-up among 5375 participants [31]. This may be explained by the fact that dietary habits deteriorate up to five years before the diagnosis of dementia presumably as an early sign of dementia [31,32].

The DO-HEALTH trial follow-up was only 3 years and pre-selection for good cognitive function may have captured a homogeneous population at relatively low risk of cognitive decline in this time period presumably derogating a possible benefit from the MIND-diet. Another source of heterogeneity between cohort studies may be the use of different tools to assess cognitive function [15].

Repeated assessments of cognitive tests can lead to practice effects in participants and a seeming improvement in cognition over time [33]. In our study, we assessed cognition four times over the DO-HEALTH study period and observed improved cognitive function over time [25]. We therefore accounted for practice effects in our sensitivity analyses using the reliable change index (RCI) proposed by Chelune et al. [27] and Perdices [28]. Notably, our results appeared to be robust when applying this correction.

Regarding inflammatory biomarkers, we did not find a significant association between adherence to the MIND-diet score and changes in blood concentrations of IL-6 or hsCRP over three years. One explanation may be the pre-selection by generally good health state and good cognitive function in DO-HEALTH [34,35]. Thus our findings do not question other studies that found higher levels of IL-6 and hsCRP in patients with mild cognitive impairment and Alzheimer’s disease [36]. Further, we may have missed the long-term relevance of inflammation in the context of MCI, as suggested by one study where individuals with higher concentrations of inflammatory markers in midlife had a higher risk of cognitive decline later in life [37]. Furthermore, other beneficial effects of the MIND-diet may be at play including the inhibition of beta-amyloid deposition [9,38].

Our study has several strengths. First, our study includes a comprehensive diet assessment at baseline and targeted adults aged 70 years and older with good cognitive function at baseline. Further, MCI was assessed based on repeated MoCA tests, which we adjusted in a sensitivity analysis for practice effects [27]. Further, our study includes generally healthy and active older adults from five European countries.

Our study also has limitations. The population of DO-HEALTH has been pre-selected based on good cognitive function, mobility, and absence of severe health events in the five years prior to inclusion. Thus, our findings are not representative of the general older adult population and are not population-based. Further, our findings are observational and cannot establish causality. Also, DO-HEALTH was not powered to detect an association of diet and MCI and the follow-up time of three years might be considered short for a change in cognitive function.

## 5 Conclusion

In summary, this study found no significant association between the MIND diet and cognitive impairment over three years in relatively healthy, community dwelling adults over 70 years in the DO-HEALTH Cohort. Moreover, no significant association was found between the adherence to the MIND diet and inflammatory markers.

## Funding

DO-HEALTH was funded by the Seventh Research Framework Program of the European Commission (Grant Agreement n°278588, PI Bischoff-Ferrari HA), and within this framework, also by the 10.13039/501100006447University of Zurich(Chair for Geriatric Medicine and Aging Research), DNP,Roche,NESTEC,Pfizer,Streuli. In addition, the current study received partial funding from EMPIRIS Foundation.

The funding/supporting organizations had no role in the design and conduct of the study, including collection, management, analysis, and interpretation of the data, as well as preparation, review, or approval of the manuscript, or decision to submit the manuscript for publication.

## Declaration of interests

The authors declare the following financial interests/personal relationships which may be considered as potential competing interests:

Heike Bischoff-Ferrari: financial support was provided by European Commission, University of Zurich, DNP, Roche, Nestec SA, Pfizer, Streuli Pharma AG, EMPIRIS Foundation; relationship with BioMed that includes paid expert testimony; relationship with Vifor Pharma Switzerland SA that includes speaking and lecture fees; relationship with OM Pharma that includes speaking and lecture fees.

Ralph C. Schimmer: honoraria for lecture/presentation from Hirslanden Hospital Group; scientific expert honoraria from Innosuisse; scientific Advisor at Hygiaso; former employee Roche Pharmaceuticals and Diagnostics.

If there are other authors, they declare that they have no known competing financial interests or personal relationships that could have appeared to influence the work reported in this paper.
