# Supplementary material for: Adherence to the MIND diet and the odds of mild cognitive impairment in generally healthy older adults: The 3-year DO-HEALTH study
Source: J Nutr Health Aging. 2024 Feb 5;28(3):100034. doi: 10.1016/j.jnha.2023.100034 (PMC12880404; doi:10.1016/j.jnha.2023.100034)
Supplement: Supplementary file 1 [file mmc1.docx]

Supplementary Material

**Adherence to the MIND diet and the odds of mild cognitive impairment in generally healthy older adults: The 3-year DO-HEALTH study**

**Roman Sager Stephanie Gaengler, Walter C. Willett, E. John Orav, Michele Mattle, Jana Habermann, Katharina Geiling, Ralph C. Schimmer, Bruno Vellas, Reto W. Kressig, Andreas Egli, Bess Dawson-Hughes, Heike A. Bischoff-Ferrari**

Table S1 Frequencies and percentages of mild cognitive impairment over the study duration of DO-HEALTH using the different cut-offs and indication of direction of change of MCI status over time compared to the previous time point.

| Outcome | Time point | MCI-staus , n (%) | Deteriorated, n (%) | Unchanged, n (%) | Improved, n (%) |
| --- | --- | --- | --- | --- | --- |
| MoCA<26 | Baseline | 775 (39.1) |  |  |  |
|  | Year 1 | 588 (32.9) | 179 (10.0) | 1328 (74.4) | 279 (15.6) |
|  | Year 2 | 539 (31.5) | 163 ( 9.5) | 1368 (80.1) | 177 (10.4) |
|  | Year 3 | 516 (30.3) | 154 ( 9.3) | 1325 (80.3) | 171 (10.4) |
| MoCA<24 | Baseline | 411 (20.7) |  |  |  |
|  | Year 1 | 300 (16.8) | 101 ( 5.7) | 1524 (85.3) | 161 ( 9.0) |
|  | Year 2 | 273 (15.9) | 96 ( 5.6) | 1504 (88.1) | 108 ( 6.3) |
|  | Year 3 | 268 (15.7) | 93 ( 5.6) | 1469 (89.0) | 88 ( 5.3) |
| MoCA-RCI<26 | Year 1 | 731 (40.9) | 156 ( 8.7) | 1517 (84.9) | 113 ( 6.3) |
|  | Year 2 | 629 (38.1) | 124 ( 7.5) | 1404 (85.1) | 121 ( 7.3) |
|  | Year 3 | 578 (35.8) | 48 ( 3.1) | 1417 (91.1) | 91 ( 5.8) |
| MoCA-RCI<24 | Year 1 | 369 (20.7) | 87 ( 4.9) | 1621 (90.8) | 78 ( 4.4) |
|  | Year 2 | 334 (20.0) | 84 ( 5.0) | 1525 (91.5) | 57 ( 3.4) |
|  | Year 3 | 286 (17.4) | 16 ( 1.0) | 1522 (95.7) | 53 ( 3.3) |
| Abbreviations: MCI- mild cognitive impairment , MoCA- Montreal cognitive assessment , RCI-Reliable change index | | | | | |
